# Honeysuckle extract relieves ovalbumin-induced allergic rhinitis by inhibiting AR-induced inflammation and autoimmunity

**DOI:** 10.1042/BSR20190673

**Published:** 2019-07-29

**Authors:** Bin Lin, Bijuan Cai, Huige Wang

**Affiliations:** 1 ENT Department, Guangzhou Hospital of Integrated Traditional and West Medicine, No. 87 Yingbin Road, Huadu District, Guangzhou 510800, Guangdong Province, China; 2 ENT Department, The First Affiliated Hospital of Shantou University Medical College, Shantou, China

**Keywords:** Allergic rhinitis (AR), Autoimmunity, Honeysuckle extract (HE), Inflammation

## Abstract

Honeysuckle has antiviral, antioxidative and anti-inflammatory properties. Allergic rhinitis (AR) is induced by immunoglobulin E (IgE)-mediated inflammatory reaction. Our study investigates whether honeysuckle extract (HE) has therapeutic effect on AR. An AR model of mice was established by ovalbumin (OVA). Hematoxylin–Eosin staining was used to assess nasal mucosa damage. Enzyme-linked immunosorbent assay (ELISA) was performed to determine serum histamine, IgE and interleukin (IL)-2, IL-4, IL-17 and interferon-γ (IFN-γ) from nasal lavage fluid. Western blot was carried out to analyze the protein level from nasal mucosa tissue. We found that HE not only decreased nasal rubbing and sneezing in AR mice, but also reduced AR-induced damage to nasal mucosa. Moreover, HE lowered the levels of serum IgE and histamine and inhibited IL-4 and IL-17 levels from AR mice but raised IL-2 and IFN-γ levels in AR-induced nasal lavage fluid. Our results also showed that HE elevated the protein levels of forkhead box P3 (Foxp3) and T-box transcription factor (T-bet) in AR-induced nasal mucosa tissue, whereas it inhibited signal transducer and activator of transcription (STAT) 3 and GATA binding protein 3 (GATA-3) protein levels. By regulating AR-induced inflammatory reaction and autoimmune response, HE also relieved OVA-induced AR. Thus, HE could be used as a potential drug to treat AR.

## Introduction

Caused by inhaled allergens, allergic rhinitis (AR) is a hypersensitivity reaction-induced, immunoglobulin E (IgE)-mediated inflammatory reaction in nasal mucosa [1,2]. It is reported that the prevalence of AR in general population was 15–20%, for example, in the Swiss population from 0.3% in 1926 to 14% in 1995 [3]; moreover, the number of Japanese AR patients also has increased, as a report showed that AR was prevailing among young Japanese [4]. Inhaled antigens were distributed in nasal mucosa by binding IgE to epithelial cells and nasal mucosal mast cells. Chemical transmission substance such as histamie and inflammatory cytokines are then released in response to such an immune response [4,5]. A series of responses stimulate sensory nerve and nasal mucosa to cause sneezing, rhinorrhea and nasal itching [4,6].

Strategies for treating AR were, for example, immunotherapy (pharmacologic) and elimination or avoidance of allergen (non-pharmacologic) [2,7]. Pharmacotherapy contains intranasal corticosteroid, oral or intranasal antihistamine and immunotherapy [8], for example, loratadine, a second-generation antihistamine, significantly relieves the symptoms of AR [9], decongestants reduce nasal congestion of AR and nasal inflammation [8]. Honeysuckle, which is a traditional Chinese herb, has a large proportion of arsenite, imethylarsinic acid and monomethylarsonic acid as its main chemical ingredients [10]. Lee et al. [11] indicated that honeysuckle inhibited dengue virus replication by decreasing let-7a, suggesting that honeysuckle had an antiviral property. Honeysuckle prevents oxidative stress damage by accumulation of leaf phenolics, bioactive compound [12], showing that honeysuckle had an antioxidative property. Moreover, honeysuckle inhibits lipopolysaccharide (LPS)-mediated inflammatory response [13–15] by interleukin (IL)-1 receptor-associated kinase (IRAK)-1/TGF-β-activated kinase (TAK)-1 pathway and decreasing pro-inflammatory factors (IL-1β, IL-6, nitric oxide (NO), tumor necrosis factor (TNF)-α and cyclooxygenase-2 (COX-2)), which indicated that honeysuckle has anti-inflammatory property. Our study investigated whether honeysuckle extract (HE) had an inhibitory effect on AR and on AR-produced inflammation and autoimmunity, according to etiopathogenesis of AR and properties of honeysuckle.

## Materials and methods

### HE preparation

HE was purchased directly from Meiherb (Dongguan, Guangdong, China). HE was dissolved in saline solution (Solarbio, Beijing, China) to form 50 mg/ml. The concentrations of HE25 and HE50 were 25mg/ml and 50mg/ml in the following experiments.

### AR model of mice and treatment

Male Balb/c mice aged 6 weeks were obtained from Animal Centre of Sun Yat-sen University (Guangzhou, Guangdong, China) and were used to establish an AR model. Untreated mice (normal mice) were regarded as control group (*n*=8), while mice made as AR model were injected with 0.5 ml mixed solution containing ovalbumin (0.5 mg/ml, OVA) (Solarbio, Beijing, China) and aluminium hydroxide (20 mg/ml) (Sigma–Aldrich, St. Louis, Missouri, U.S.A.) by intraperitoneal injection (ip) and fed for 7 and 14 days. OVA and aluminium hydroxide were dissolved in physiological saline (Solarbio, Beijing, China), and the solution was sterilized by filtration (Thermo Scientific, Waltham, Massachusetts, U.S.A.). Mice were divided into four groups (*n*=8), which were AR group, Loratadine group, HE 25 group and HE50 group. At the 15th day, local stimulation was performed on all mice by adding OVA (40 mg/ml in physiological saline) into double nasal cavity during 10 days. In Loratadine group, mice were administrated Loratadine (2 mg/kg) (Sigma–Aldrich, St. Louis, Missouri, U.S.A.) by irrigation before receiving daily local stimulation. In HE25 group, mice were administrated HE (25 mg/kg) (Meiherb, Dongguan, Guangdong, China) by irrigation before receiving daily local stimulation, while mice in HE50 group were administrated HE (50 mg/kg) by irrigation. All mice were placed under 12-h lighting (7:00–19:00) and 12-h dark (19:00–7:00) cycle in an air environment (temperature: 23 ± 2°C, relative humidity: 55 ± 5%). Animal experimental operations in our study were performed in accordance with Guide for Animal Care and Use of Guangzhou Hospital of Integrated Traditional and Western Medicine. Animal experiments were approved by Ethics Committees and Health Authorities of Guangzhou Hospital of Integrated Traditional and Western Medicine (No. TW201803019E). The mice models of AR and the AR improvement by HE were evaluated for physiological and biochemical parameters [16,17].

### Physiological parameters

AR physiological symptoms were observed according to count of the number of rubbing and sneezing in a randomized blind manner for 40 min following nasal challenge [18]. Rubbing is characterized by an external perinasal scratch with forelimbs of animal and sneezing is characterized by an explosive expiration after a deep inspiration [18].

### Biochemical parameters

AR biochemical symptoms were detected hypersensitivity mediator, such as histamine, and inflammatory mediators such as lgE, IL-2, IL-4, IL-17 and interferon-γ (IFN-γ). In addition, we could observe the eosinophils (EOS) infiltration. The EOS was observed as often being round or elliptical in shape, as well as having a double-leaf nucleus [19].

### Enzyme-linked immunosorbent assay

Blood was extracted from mice after they were killed, and serum was obtained by centrifuging the blood collected (Cence, Changsha, Hunan, China). Histamine and IgE were determined by using histamine and IgE Enzyme-linked immunosorbent assay (ELISA) kit (Jiancheng Bioengineering Institute, Nanjing, Jangsu, China) according to the instructions.

Nasal lavage fluid samples were collected from mice by washing their noses at the end of animal experiment. IL-2, IL-4, IL-17 and IFN-γ from nasal lavage fluid were detected by IL-2, IL-4, IL-17 and IFN-γ ELISA kit (Invitrogen, Carlsbad, California, U.S.A.), respectively.

### Hematoxylin–Eosin staining

Nasal mucosa was extracted from mice after the mice had been executed by decapitation. Nasal mucosa was fixed to paraffin section, dehydrated and paraffin-embedded. Hematoxylin dye (Solarbio, Beijing, China) was used to stain the sections for 5 min, and the section was then washed by water. Next, ammonia was used to soak the sections, which were then washed by water. The sections were stained by 1% Eosin dye (Solarbio, Beijing, China) and then washed by water.

### Western blot

Nasal mucosa tissues were cut and centrifuged to extract proteins using TRIzol reagent (Invitrogen, Carlsbad, California, U.S.A.). BCA kit (Thermo Scientific, Waltham, Massachusetts, U.S.A.) was used to measure protein concentration, and protein ladder (Invitrogen, Carlsbad, California, U.S.A.) was separated from protein by SDS/PAGE. Next, the proteins were transferred to PVDF membranes (Sigma–Aldrich, St. Louis, Missouri, U.S.A.), which were blocked by 5% albumin. The protein membranes were incubated first with primary antibody (Table 1) was used to incubate at 4°C for 12 h and then with secondary antibody (ab7090, Abcam, Cambridge, Massachusetts, U.S.A.) at 25°C for 3 h. Primary antibody and secondary antibody were dissolved in TBST solution (Solarbio, Beijing, China) following the instructions. ECL kit (Sigma–Aldrich, St. Louis, Missouri, U.S.A.) was used to stain the membranes in the dark. The protein flaser were taken by fluorescence imaging system (CliNX, Shanghai, China).

**Table 1:** Primary antibodies in Western blot

Name	Molecular weight	Item number	Manufacturer
IL-17	18 kDa	ab79056	Abcam, Cambridge, Massachusetts, U.S.A.
STAT-3	88 kDa	ab68153	Abcam, Cambridge, Massachusetts, U.S.A.
T-bet	58 kDa	ab53174	Abcam, Cambridge, Massachusetts, U.S.A.
Foxp3	73 kDa	ab215206	Abcam, Cambridge, Massachusetts, U.S.A.
GATA-3	48 kDa	ab106625	Abcam, Cambridge, Massachusetts, U.S.A.
GAPDH	36 kDa	ab181602	Abcam, Cambridge, Massachusetts, U.S.A.

Abbreviations: Foxp3, forkhead box P3; GATA-3, GATA binding protein 3; STAT, signal transducer and activator of transcription; T-bet, T-box transcription factor.

### Quantitative polymerase chain reaction

RNAs were extracted from nasal mucosa tissue by RNA purification kit (Invitrogen, Carlsbad, California, U.S.A.). cDNAs were synthesized by extracted RNAs using reverse transcription kit (Applied Biosystems, Carlsbad, California, U.S.A.). RNAs were amplified using cDNAs, primers (Table 2) and transcription kit (Invitrogen, Carlsbad, California, U.S.A.) at 95°C for 30 s, at 55°C for 45 s and at 75°C for 30 s for 55 cycles. All operations were conducted following the instruction. Primers were synthesized by Invitrogen (Carlsbad, California, U.S.A.).

**Table 2:** Primers in quantitative polymerase chain reaction

Name	Forward (5′–3′)	Reverse (5′–3′)
IL-17	CTGATCAGGACGCGCAAAC	TCGCTGCTGCCTTCACTGTA
STAT-3	TTGCCAGTTGTGGTGATC	AGACCCAGAAGGAGAAGC
T-bet	TCCCATTCCTGTCCTTCA	GCTGCCTTCTGCCCTTTC
Foxp3	CTGGATGAGAAAGGCAAGG	AAAGTGGATACGGTGGGAA
GATA-3	TCTCACTCTCGAGGCAGCATGA	GCTACCATCTCGCCGCCACAG
β-actin	AAGTGTGACGTTGACATCCG	TCTGCATCCTGTCAGCAATG

Abbreviations: Foxp3, forkhead box P3; GATA-3; GATA binding protein 3; STAT, signal transducer and activator of transcription; T-bet, T-box transcription factor.

### Statistical analysis

Western blot, Hematoxylin–Eosin staining, ELISA and quantitative polymerase chain reaction (qPCR) were performed for at least three times. All data were analyzed by F test using SPSS 19.0 (IBM, Armonk, New York, U.S.A.) and shown as Mean ± Standard Deviation (SD). *P*<0.05 indicated a significant difference.

## Results

### HE decreases symptoms in AR mice

**Figure 1:**
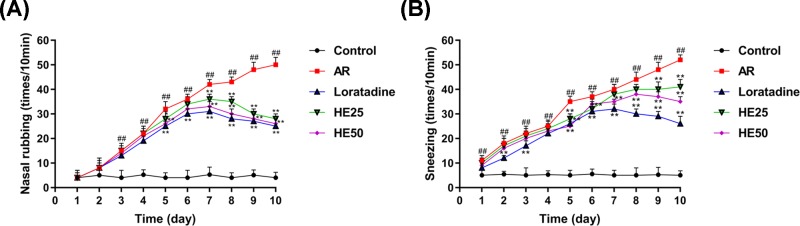
HE reduces the frequency of nasal rubbing and sneezing in AR mice Male Balb/c mice were used to establish an AR model by OVA. Untreated male Balb/c mice (normal mice) (n=8) were regarded as control group. AR mice were divided into four groups (n=8) and treated by HE and Loratadine in each group before local stimulation. The frequency of nasal rubbing (**A**) and sneezing (**B**) were recorded. Loratadine group was regarded as drug positive group. Values were shown as mean ± SD, and data were analyzed using F test (##vs Control group and ** vs AR group; ** = ## = *P*<0.01).

AR significantly increased nasal rubbing (Figure 1A) and sneezing (Figure 1B) in mice. Loratadine (therapy group), a second-generation antihistamine drug, has been used to treat AR in clinical practice [2,20]. Loratadine was found to reduce AR-induced nasal rubbing (Figure 1A) and sneezing (Figure 1B). HE also reduced nasal rubbing (Figure 1A) and sneezing (Figure 1B) in AR mice, and a higher concentration of HE produced more obvious effect.

**Figure 2:**
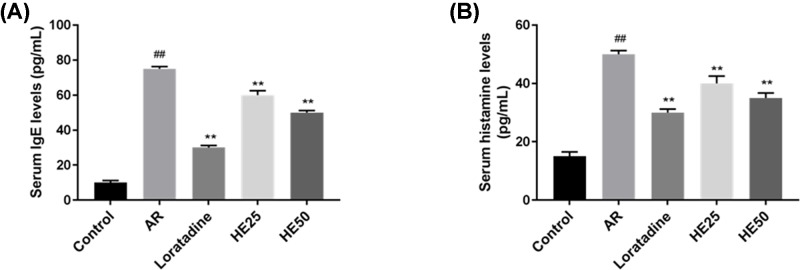
HE reduces serum levels of IgE and histamine in AR mice IgE (**A**) and histamine (**B**) from blood samples at the end of animal experiment were determined by ELISA. Loratadine group was regarded as drug positive group. Values were shown as mean ± SD, and data were analyzed using F test (## vs Control group and ** vs AR group; ** = ## = *P*<0.01).

### HE lowers the levels of serum IgE and histamine in AR mice

AR significantly raised the levels of serum IgE (Figure 2A) and histamine (Figure 2B), compared with control group, however, HE lowered the levels of serum IgE (Figure 2A) and histamine (Figure 2B) in AR mice, and a higher concentration of HE produced more obvious effect. Loratadine group had the lowest levels of serum IgE (Figure 2A) and histamine (Figure 2B), compared with AR group and HE groups (HE25 and HE50). HE groups had a significant difference from AR group (*P*<0.01).

### Effects of HE on the levels of IL-2, IL-4, IL-17 and IFN-γ in nasal lavage fluid from AR mice

**Figure 3:**
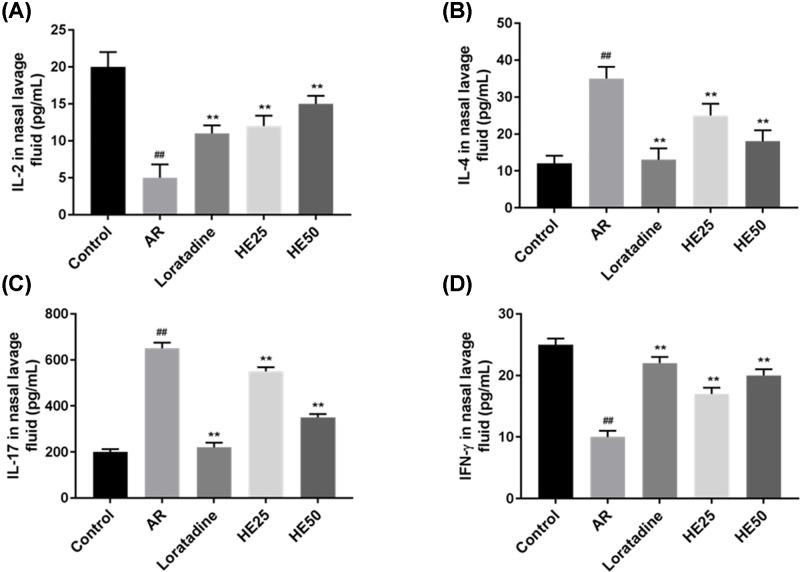
Effects of HE on the levels of IL-2, IL-4, IL-17 and IFN-γ from nasal lavage fluid of AR mice Nasal lavage fluid was collected at the end of animal experiment. ELISA was performed to detect the contents of IL-2, IL-4, IL-17 and IFN-γ. HE increased the contents of IL-2 (**A**) and IFN-γ (**D**) in AR-induced nasal lavage fluid. HE decreased the contents of IL-4 (**B**) and IL-17 (**C**) in AR-induced nasal lavage fluid. Loratadine group was regarded as drug positive group. Values were shown as Mean ± SD, and data were analyzed using F test (## vs Control group and ** vs AR group; ** = ## = *P*<0.01).

AR lowered IL-2 (Figure 3A) and IFN-γ (Figure 3D) levels from nasal lavage fluid. However, Loratadine and HE raised the levels of IL-2 (Figure 3A) and IFN-γ (Figure 3D) in AR-induced nasal lavage fluid. AR increased IL-4 (Figure 3B) and IL-17 (Figure 3D) levels from nasal lavage fluid. Loratadine and HE decreased the levels of IL-2, IL-4 (Figure 3B) and IL-17 (Figure 3D) in AR-induced nasal lavage fluid, and the levels were the lowest in Loratadine group, compared with two HE groups (HE25 and HE50) (Figure 3B,C). The inhibitory effect increased as HE concentration went up. HE groups had a significant difference from AR group (*P*<0.01).

### HE decreases AR-induced nasal damage

**Figure 4:**
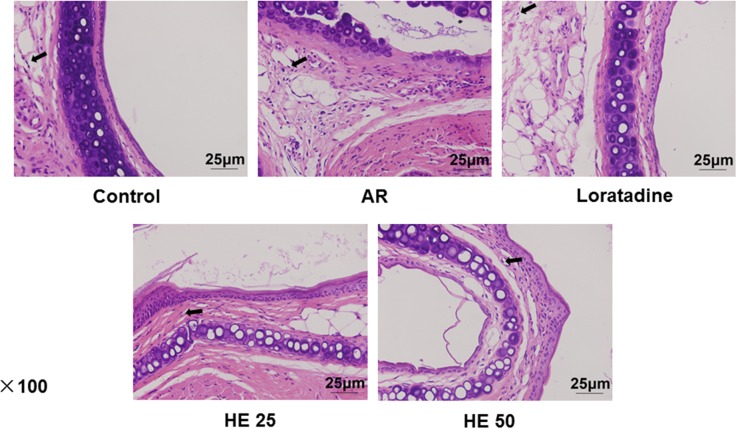
HE relieves AR-induced nasal damage The nasal damage showed as tissue edema, small vessel dilatation and gland hyperplasia, with EOS infiltration. Nasal mucosa tissues from mice in five groups (control, AR, Loratadine, HE25 and HE50) were made to paraffin section, and Hematoxylin and Eosin were used to stain the sections. Loratadine group was regarded as drug positive group.

Mice in the control group had hardly tissue edema, small vessel dilatation and gland hyperplasia, with a small amount of EOS infiltration, which was considered to be nasal damage. Compared with AR which caused seriously nasal damage (Figure 4) we found that Loratadine and HE decreased AR-induced nasal damage (Figure 4), which reduced hyperemia, edema and EOS infiltration. Loratadine had a better efficacy, compared with HE, and a higher concentration of HE produced more obvious effect (Figure 4).

### Effects of HE on the levels of IL-17, signal transducer and activator of transcription, T-box transcription factor, forkhead box P3 and GATA binding protein 3 in nasal mucosa tissue of AR mice

**Figure 5:**
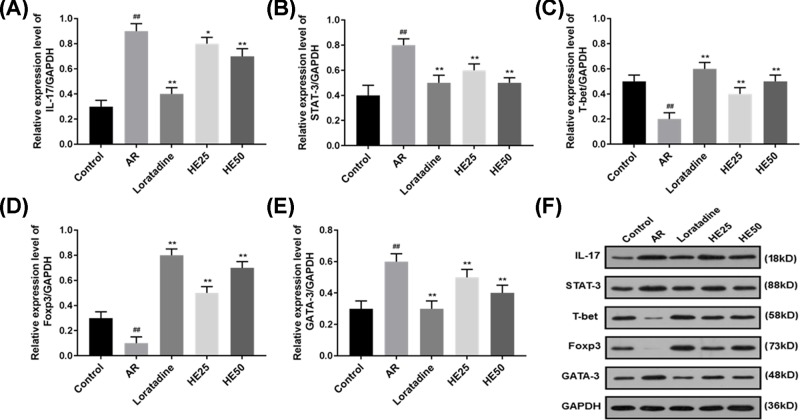
Effects of HE on the protein levels of IL-17, STAT3, T-bet, Foxp3 and GATA-3 from nasal mucosa tissue of AR mice Protein was extracted from nasal mucosa tissue. (**A**–**F**) The protein levels of IL-17, STAT 3, GATA-3, T-bet and Foxp3 were determined by Western blot. HE decreased the protein levels of IL-17 (A), STAT-3 (B) and GATA-3 (E) in AR-induced nasal mucosa tissue. HE increased the protein levels of T-bet (C) and Foxp3 (D) in AR-induced nasal mucosa tissue. Loratadine group was regarded as drug positive group. Values were shown as Mean ± SD, and data were analyzed using F test (## vs Control group and * vs AR group; * *P*<0.05 and ** = ## = *P*<0.01).

**Figure 6:**
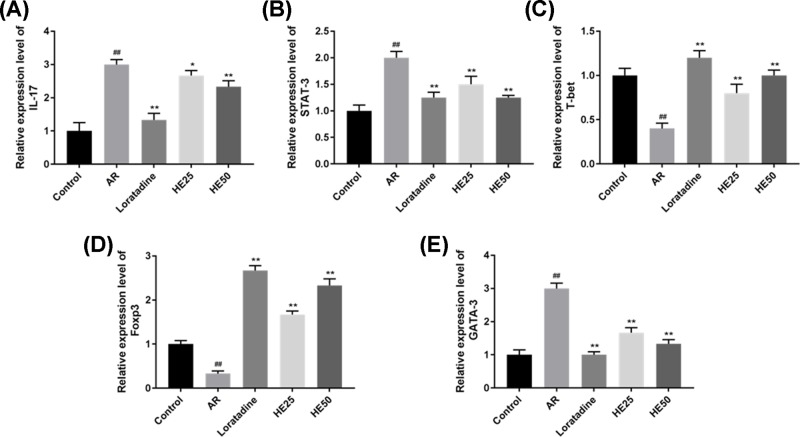
Effects of HE on the mRNA expression of IL-17, STAT3, T-bet, Foxp3 and GATA-3 from nasal mucosa tissue of AR mice mRNA was extracted from nasal mucosa tissue. The mRNA levels of IL-17, STAT 3, GATA-3, T-bet and Foxp3 were determined by qPCR. HE lowered the relative mRNA levels of IL-17 (**A**), STAT-3 (**B**) and GATA-3 (**E**) in AR-induced nasal mucosa tissue. HE raised the relative mRNA levels of T-bet (**C**) and Foxp3 (**D**) in AR-induced nasal mucosa tissue. Loratadine group was regarded as drug positive group. Values were shown as Mean ± SD, and data were analyzed using F test (## vs Control group and * vs AR group; * *P*<0.05 and ** = ## = *P*<0.01).

HE and Loratadine affected IL-17 level nasal mucosa tissue of AR mice (Figures 5A,F and 6A), which was similar to the level of IL-17 in nasal lavage fluid from AR mice. AR raised the levels of signal transducer and activator of transcription (STAT) 3 and GATA binding protein 3 (GATA-3) of nasal mucosa tissue; however, HE and Loratadine lowered AR-induced STAT3 and GATA-3 level in nasal mucosa tissue (Figures 5B,E,F and 6B,E). The levels of T-box transcription factor (T-bet) and forkhead box P3 (Foxp3) in nasal mucosa tissue were lowered by AR, however, HE and Loratadine could increase AR-induced T-bet and Foxp3 level in nasal mucosa tissue (Figures 5C,D,F and 6C,D). HE groups had a significant difference from AR group (*P*<0.01 or *P*<0.05).

## Discussion

In our study, HE relieved the symptoms of AR such as sneezing and nasal itching, suggesting that HE had therapeutic effect on AR. Besides, a higher concentration of HE had a better effect on AR. AR patients tend to have a higher IgE in response to allergens, according to etiopathogenesis of AR [21]. The cause of allergic reactions is a result of histamine-producing mast cells causing eosinophil phagocytic cells, a process associated with alloimmunization [22,23]. IgE and histamine are biochemical indicators in evaluating drug efficacy [24,25]. Loratadine, treated as drug positive group and is used in the treatment of AR [9]. However, long-term or high-dosage use of loratadine in AR can cause undesirable side effects, for example anaphylactic reactions [26] and sedation [27]. HE had a therapeutic activity for AR, and a higher HE concentration had a better therapeutic effect on AR. However, to understand the mechanism by which HE affected AR, the mechanism of inflammation and immunization was investigated using Loratadine, a drug for treating AR [9] and treated as drug positive group.

AR model of mice was observed to have inflammation [28], and patients with AR also had inflammation, according to clinical analysis [29]. Ingested inflammatory factor (IL-25) aggravates OVA-induced AR by p38/NF-κB signaling pathway [30], suggesting that inflammatory reaction plays an important role in AR. From the perspective of nosogenesis, IgE-mediated inflammation is a significant component of allergic mechanism [31]. INF-γ, IL-2, IL-17 and IL-4 are pro-inflammatory cytokines [32–35]. IL-17 and IL-4 regulate pro-inflammatory genes (IL-19, IL-20 and IL-6) via NF-κB signaling pathway [35–37], and HE decreased AR-induced inflammatory reaction via IL-17/IL-4 pathway. However, patients with intermittent AR during non-pollen season have higher IL-2 and INF-γ expressions [38]. Chevalier et al. [39] pointed out that inflammation can trigger autoimmunity by reducing IL-2. Decreased IL-2 by HE may be associated with the balance between inflammation and autoimmunity. In line with above reports, we could find that HE50 had a similar effect on up-regulation of the levels of IL-2 and IFN-γ and down-regulation of IL-4 and IL-17, which presented an anti-inflammatory effect.

Allergen can cause autoimmune disease such as scleroderma, arthritis and rhinitis [40,41]. Immunotherapy is used to treat AR, and immunotherapy of AR affects B-cell differentiation and decreases B-cell sensibility [42]. Autoimmunity risk of AR can be regulated by genes such as *FCRL3* [43]. T-bet expressed in B cell is regarded as a transmitter of autoimmunity during antiviral responses in mammals [44]. However, reducing T-bet in addition to increased STAT-6 and GATA-3 induces allergic responses in humans [45]. Moreover, AR patients have higher expressions of Foxp3 and GATA-3 [46]. T-bet, GATA-3 and Foxp3 are regarded as bio-indicators in evaluating therapeutic efficacy for AR [47]. Innate immune pro-inflammatory responses to immunological disease are associated with STAT-3 mutation [48]. In the present study, AR causes abnormal STAT-3 expression, and HE50 significantly decreased STAT-3 mutation. Accordingly, HE could increase T-bet, Foxp3 expressions and reduce the expressions of GATA-3 and IL-17.

There are still some limitations, for example, the lack of more comprehensive scoring criteria and histological evaluation in this mouse model of AR and lacking of immunohistochemical analysis of signaling pathway proteins changes in nasal mucosa. We are planning to take out a further in-depth study.

## Conclusion

In conclusion, HE had a therapeutic efficacy for treating AR by assessing the symptoms of AR in AR model of mice. Moreover, HE relieved AR by decreasing AR-induced inflammation and autoimmunity. Thus, our study provided basic evidence that HE could be used as a drug for AR.

## Abbreviations

AR: allergic rhinitis
ELISA: enzyme-linked immunosorbent assay
EOS: Eosinophils
Foxp3: forkhead box P3
GATA-3: GATA binding protein 3
HE: honeysuckle extract
IFN-γ: interferon-γ
IgE: immunoglobulin E
IL: interleukin
OVA: ovalbumin
STAT: signal transducer and activator of transcription
T-bet: T-box transcription factor
TBST: Tri-Buffered Saline Tween20
